# Bilateral Continuous Femoral Nerve Blocks as an Opioid-Sparing Strategy for Refractory Lower Extremity Pain in Adult Sickle Cell Crisis: A Case Report

**DOI:** 10.3390/reports9020110

**Published:** 2026-04-02

**Authors:** Thomas Renfrew, Thomas Oh, Derek Chung, Yuri C. Martins, Hamed Sadeghipour

**Affiliations:** Department of Anesthesiology and Critical Care, SSM Health Saint Louis University Hospital, Saint Louis, MO 63104, USAthomas.oh@slucare.ssmhealth.com (T.O.); yuri.chavesmartins@slucare.ssmhealth.com (Y.C.M.)

**Keywords:** femoral nerve catheter, regional anesthesia, sickle cell disease, vaso-occlusive crisis

## Abstract

**Background and Clinical Significance:** Sickle cell vaso-occlusive crisis (VOC) may present with severe refractory pain that is difficult to control despite guideline-directed multimodal therapy and high-dose opioids. **Case Presentation:** We report an adult with VOC and severe, opioid-refractory anterior thigh and leg pain who was treated with bilateral, ultrasound-guided continuous femoral nerve catheters (0.5% bupivacaine bolus per side followed by infusion of 0.2% ropivacaine at 5 mL/h each). Twenty-four-hour opioid use decreased by 76% from 44 mg intravenous hydromorphone (880 MME) before block placement to 10.4 mg (208 MME) after catheter initiation. Pain scores declined significantly from 10/10 to 3/10, facilitating mobilization and expediting discharge of the patient. No local anesthetic systemic toxicity occurred, and transient quadriceps weakness was managed with fall-risk precautions. IRB approval for this case report was waived per our institution policy. **Conclusions:** In select adults with VOC and predominant anterior thigh/leg pain, bilateral continuous femoral nerve catheters may provide rapid analgesia and substantial opioid-sparing benefits as part of multidisciplinary care. These findings are hypothesis-generating and support prospective evaluation of continuous peripheral nerve block strategies in VOC.

## 1. Introduction and Clinical Significance

Sickle cell disease (SCD) is an inherited hemoglobinopathy in which a β-globin mutation produces hemoglobin S. When deoxygenated, hemoglobin S polymerizes, distorting erythrocytes into rigid “sickled” cells. These cells promote microvascular obstruction and ischemia–reperfusion injury that may lead to vaso-occlusive crisis (VOC), the leading cause of hospitalization in SCD patients, and a driver of high opioid exposure despite guideline-directed multimodal therapy [1,2,3]. In the United States, the burden of these VOCs is substantial. Approximately 230,000 SCD-related hospital admissions occur annually, with VOCs being attributed as either a primary or secondary diagnosis in 78.3% of these hospitalizations [4].

Pain control with opioids is usually the first-line treatment for adults experiencing VOC. Guideline-directed multimodal strategies, such as non-steroidal anti-inflammatory drugs, gabapentinoids, corticosteroids, ketamine, and mind–body therapies, may be added, but the American Society of Hematology notes low certainty in the supporting evidence for effectiveness of each of these recommendations [5].

Locoregional anesthesia (LRA) has also been proposed as an opioid-sparing adjunct for refractory VOC, and it has shown great promise in adults and pediatric populations [6]. While pediatric case reports and case series describe both single-injection and continuous peripheral nerve blocks, published adult data remain limited [7,8]. However, pediatric cases account for only 19.2% of total hospitalizations due to SCD complications, meaning that this promising technique has not been studied in the vast majority of the contexts in which VOCs occur.

We report an adult with opioid-refractory VOC whose pain was managed with bilateral, ultrasound-guided continuous femoral nerve catheters, resulting in marked analgesia and opioid reduction. Within 24 h, opioid use decreased by ~76%, from 44 mg intravenous (IV) hydromorphone (880 MME) pre-block to 10.4 mg (208 MME) post-block, supporting the feasibility and potential value of regional pain block with continuous peripheral nerve catheters as an adjunct in selected adults with VOC.

## 2. Case Presentation

A written consent was obtained from the patient for publication of this case report. A 36-year-old, 134 kg, African American man with SCD presented to the emergency department with a VOC characterized by severe bilateral lower-extremity pain. His SCD history included frequent VOCs and prior episodes of acute chest syndrome, avascular necrosis of both humeral heads, pulmonary hypertension, intermittent priapism, and multiple pulmonary emboli. Initial emergency department evaluation was otherwise unremarkable. He was then admitted for intravenous (IV) fluids and analgesia treatment. His home pain regimen consisted of morphine 30 mg twice daily (BID) and hydrocodone-acetaminophen 10/325 mg every 4 h (q4h) as needed (PRN). After admission, he was started on morphine controlled-release 45 mg BID, hydrocodone-acetaminophen 10/325 mg q6h PRN, hydromorphone 3 mg q3h PRN, methocarbamol 1000 mg daily, and gabapentin 300 mg three times daily (TID). Due to persistent severe pain, therapy was escalated. On hospital day 6 a hydromorphone patient-controlled analgesia (PCA) was initiated (basal 0.2 mg/h; 0.2 mg demand every 10 min PRN; 1.4 mg/h maximum hourly rate), replacing morphine and hydrocodone-acetaminophen. PCA output was documented based on total drug consumption within each 8 h period and per 24 h, documented by both EMR and Nursing Staff. Despite titration over subsequent days to basal 1.0 mg/h, 0.8 mg demand every 12 min, and 5.0 mg/h maximum, analgesia remained inadequate. He additionally received a red blood cell exchange transfusion which reduced his pain only for a few hours. He also reported constipation and drowsiness, consistent with opioid adverse effects, prompting efforts to reduce opioid exposure.

Due to the refractory nature of the patient’s pain despite guideline-directed therapy, the Regional Anesthesia and Acute Pain Service was consulted. After discussion with the primary team and shared decision-making with the patient, we proceeded with bilateral continuous femoral nerve blockade. Risk benefit analysis revealed this technique as the optimal procedure in this case because of the technical accessibility of the procedure given the patient’s limited range of motion secondary to pain, its anatomic specificity to the patient’s pain pattern, and the bilateral nature of the patient’s pain.

Following skin antisepsis, 6 mL of 1% lidocaine was infiltrated subcutaneously. Under ultrasound guidance with an in-plane approach, an 18-gauge Tuohy needle was advanced towards the femoral nerve. After negative aspiration, a single-injection block was performed using 25 mL of 0.5% bupivacaine, 4 mL of 2% lidocaine, and 1cc of 4 mg/cc of dexamethasone for a total of 30 cc; with intermittent repositioning and hydro dissection that were used to optimize spread, which was visualized sonographically. This dosage was decided upon in accordance with our pharmacy and hospital guidelines and was determined to be below the maximum tolerated dose for this patient’s weight. After the initial block, a 20-gauge, 61 cm spiral closed-tip catheter (Contiplex^®^ Echo, B. Braun, Bethlehem, PA, USA) was advanced 3 cm beyond the needle tip, the needle was removed, and the catheter was secured in place. The same procedure was performed on the contralateral side. The catheters were connected to two 600 mL elastomeric pumps containing 0.2% ropivacaine (ON-Q^®^ PainBuster^®^, Avanos Medical, Alpharetta, GA, USA) and were set at a basal rate of 5 mL/h each, per departmental protocol and confirming it with our pharmacology colleagues. The patient tolerated the procedure without any complication. Transient quadriceps weakness was initially present following catheter placement. Fall-risk mitigation strategies were discussed with the patient and the primary team, and ambulation was limited until further assessment by physical therapy (PT) team. At 24 h post-procedure, upon the return of the motor function, thorough assessment and physical exam by the pain and PT team, it was determined that return to ambulation under strict supervision would be safe. The patient was initially restricted to ambulation within his own room, but within 48 h he was able to ambulate the entirety of a 90-foot hallway with PT.

Twenty hours later, the pain scores improved significantly from 10 to 3 on a 10-point numerical rate scale, which was sustained throughout the treatment and represented the most substantial relief since admission, and the patient described it as a “game changer”. Within 24 h of catheter initiation, opioid use decreased by ~76%, from 44 mg IV hydromorphone (880 MME) to 10.4 mg IV hydromorphone (208 MME). The hydromorphone PCA was further down-titrated and subsequently discontinued over ten days following the block and catheter placement, with breakthrough analgesia managed using oxycodone 15 mg q4h PRN and IV hydromorphone 4 mg q3h PRN. Table 1 summarizes the changes in VAS pain scores and opioid consumption as related to key interventions. The left catheter was accidentally removed by the patient on post-procedure day four and was replaced the same day in similar fashion; it was ultimately removed on day nine (from the initial block) without further issues. The right catheter was removed after an uncomplicated six-day course. The patient was subsequently discharged on hydrocodone-acetaminophen 10/325 mg q4h PRN and morphine controlled-release 60 mg BID, with an outpatient plan to taper morphine by ~10% per week toward his pre-admission dose of 30 mg BID as tolerated.

## 3. Discussion

Regional anesthetic techniques have emerged as a conditional, guideline-supported option for opioid-refractory pain in VOC, with most reports to date involving pediatric patients. While pediatric case reports and case series describe both single-injection and continuous peripheral nerve blocks, published adult data remain limited [7,8]. However, this technique has gone largely unstudied in adult populations, which make up the vast majority of those presenting with VOC pain. This case adds to the literature by describing successful analgesia with bilateral, ultrasound-guided continuous femoral nerve catheters placement in an adult with VOC whose pain persisted despite optimized medical therapy.

The clinical impact was both symptomatic and quantitative. Pain scores declined rapidly, constipation and sedation improved, and discharge planning advanced once systemic opioids were reduced. These findings underscore the potential opioid-sparing role of continuous peripheral nerve blocks in carefully selected adults with VOC.

Choice of block must balance analgesic coverage and motor function. Femoral catheters provide robust coverage for anterior thigh and knee pain but can cause quadriceps weakness [9]. In our patient this was transient and mitigated by fall-risk precautions and assisted ambulation. Alternative approaches (e.g., fascia iliaca, adductor canal, or lumbar plexus techniques) may offer differing motor-sparing profiles and should be individualized to pain distribution and rehabilitation goals [10,11]. Compared with single-injection blocks, continuous catheters can extend analgesia and reduce rescue opioids in postoperative populations [12]; whether these advantages generalize to adult VOC requires prospective evaluation. Aseptic technique, site checks, documentation of coagulation status, monitoring/availability of lipid emulsion for local anesthetic systemic toxicity and other standardized safety practices are essential, particularly when bilateral catheters and higher total local-anesthetic doses are used.

This report has the following limitations: it is a single case with potential selection and co-intervention confounding (e.g., timing of transfusion and multimodal analgesics). Outcomes such as length of stay, readmissions, function, and adverse events warrant assessment in larger adult cohorts. The next practical steps include prospective registries or pragmatic trials comparing continuous catheters, single-injection techniques, and best medical therapy stratified by pain location and motor-sparing goals. Finally, earlier involvement of the Acute Pain Service may streamline patient selection, standardize care pathways (including fall-prevention protocols), and potentially shorten hospitalizations.

In summary, continuous bilateral femoral nerve blockade offered effective analgesia and a substantial reduction in systemic opioid exposure for an adult with opioid-refractory VOC involving the lower extremities. As concerns about opioid-related harms and undertreatment of SCD pain persist, continuous peripheral nerve catheters merit further systematic study as a feasible adjunct to multimodal care in selected adult patients.

## 4. Conclusions

This case demonstrates that bilateral, continuous femoral nerve catheters can serve as an effective adjunct for adults with VOC whose pain is refractory to guideline-directed multimodal therapy. The intervention provided rapid analgesia, enabled opioid sparing, and facilitated discharge. While a single case cannot establish generalizability, these findings support the feasibility and potential clinical value of continuous peripheral nerve blockade in selected adults with VOC and justify prospective studies to evaluate efficacy, safety, functional recovery, and comparative effectiveness versus alternative regional techniques and best medical therapy.

## Figures and Tables

**Table 1:** Changes in pain severity and opioid consumption in relation to key interventions during hospitalization.

Key Intervention	PCA Initiated	Exchange Transfusion	Bilateral Femoral Nerve Blockade Followed by Catheter Placement	Twenty-Four Hours Following Catheter Placement	Femoral Catheter Removal Followed by Discharge
**VAS Pain Score (0–10)**	10	10	3	3	1
**Opioid Consumption (MME/day)**	880	880	208	208	180

Table 1: Association between key clinical interventions, visual analog scale (VAS) pain scores, and daily opioid consumption (MME/day) during hospitalization. A significant pain reduction and opioid-sparing effect is demonstrated following bilateral continuous femoral nerve catheter placement and maintained following their removal.

## Data Availability

The original data presented in the study are included in the article, further inquiries can be directed to the corresponding author.

## Abbreviations

The following abbreviations are used in this manuscript:

SCD	Sickle Cell Disease
VOC	Vaso-Occlusive Crisis
LRA	Local Regional Anesthesia
